# Re-description of the assassin bug species *Pygolampisstriata* Miller, 1940 with new distributional records from Japan and Indonesia (Heteroptera, Reduviidae, Stenopodainae)

**DOI:** 10.3897/BDJ.9.e63695

**Published:** 2021-08-04

**Authors:** Kyosuke Okuda

**Affiliations:** 1 CTI REED Co., Ltd., Kamikisaki1-14-6, Urawa-ku, Saitama-shi, Saitama 330-0071, Japan CTI REED Co., Ltd. Kamikisaki1-14-6, Urawa-ku, Saitama-shi, Saitama 330-0071 Japan; 2 Saitama Museum of National History (External researcher: Animal), Nagatoro 1417-1, Nagatoro, Saitama 369-1305, Japan Saitama Museum of National History (External researcher: Animal) Nagatoro 1417-1, Nagatoro, Saitama 369-1305 Japan

**Keywords:** Heteroptera, re-description, Reduviidae, Japan, Indonesia, Stenopodainae, *
Pygolampis striata
*

## Abstract

**Background:**

*Pygolampisstriata* Miller, 1940 was previously described, based on a single male specimen. However, there are no records of the species since then. The females and nymphs were not described and knowledge about their habitat is insufficient.

**New information:**

This is the first record of the assassin bug *Pygolampisstriata* Miller, 1940 from Japan and Indonesia. Here, this species has been re-described and, for the first time, the female has been described. The species was collected from the surfaces of dried Poaceae grasslands using the "Gasa-Gasa collecting method".

## Introduction

The subfamily Stenopodainae (Hemiptera: Heteroptera: Reduviidae) comprises more than 720 species in 115 genera worldwide and is primarily found in the Tropics (Ishikawa and Miyamoto 2012, Weirauch et al. 2014). Almost all of the members have been reported to have a brown cryptic colour and appear to be closely associated with soil. Most species are attracted by light and have been collected with lights or using light traps, but little is known of their biology (Schuh and Weirauch 2020). In the Neotropical Region, the Stenopodainae fauna have been relatively well documented (Gil-Santana et al. 2015). In contrast, the study of Asian Stenopodainae fauna has long been neglected (Chen et al. 2020).

*Pygolampis* Germar, 1817 is the second largest genus in the subfamily Stenopodainae and contains 92 species worldwide. Ethiopian and Oriental Regions are especially species-rich, with approximately 74 known species (Gupta and Kauntey 2007, Ishikawa and Miyamoto 2012, Maldonado 1990, Swanson and Chordas 2018, Tomokuni and Cai 2003). This genus can be distinguished from other genera in the subfamily by the following characters: lack of spines on its anterior femora and first visible labial segment longer than the second and third segments combined (Tomokuni and Cai 2003). In Japan, two species, *P.bidentata* (Goeze, 1778) and *P.foeda* Stål, 1859, have been recorded. Additionally, some unidentified species have also been reported (Ishikawa and Miyamoto 2012, Ishikawa 2016, Komatsu 2016, Okuda 2020, Tanaka et al. 2013). I conducted field surveys and found one undetermined species. After careful observation, I identified the species as *P.striata* Miller, 1940, based on morphological characters from the original description (Miller 1940). This species was described, based on a single male specimen. Therefore, females and nymphs have not been described and knowledge about their biology is insufficient. In the present study, I re-describe the male and describe the female of this species for the first time.

## Materials and methods

Dried specimens were used, their morphological characteristics and genital structures were observed and measured under a stereomicroscope (Olympus SZ40, Olympus, Tokyo), equipped with a micrometer. For easier observation, male and female genitalia were soaked in hot 10% potassium hydroxide (KOH) solution for approximately 10 minutes to clear the soft tissues. Photographs of specimens were taken using a single-lens reflex camera (Canon 7D Mark II, Canon, Tokyo), equipped with a Canon macro lens EF 100 mm and MP-E 65 mm. All morphological terminology used herein follows Davis (1966), Rédei and Tsai (2011) and Weirauch et al. (2014).

Depositories of the related specimens are abbreviated as follows:

- ELTUA - Laboratory of Entomology, Faculty of Agriculture, Tokyo University of Agriculture, Atsugi, Japan;
- NIAES - National Institute for Agro-Environmental Sciences, Tsukuba, Japan;
- CBM - Natural History Museum and Institute, Chiba, Japan
- PCKO - Kyosuke Okuda private collection, Saitama, Japan.

## Taxon treatments

### Pygolampis striata

Miller, 1940

A8887A15-197B-5A2B-8AA5-7F43BB0A6669

Pygolampis striata : Miller 1940: 455, New species, description and figures (Fig. 25; 10–12); Maldonado 1990: 532, catalogue; Tomokuni and Ishikawa 2008: 374, checklist.
Pygolampis sp.: Ishikawa and Miyamoto 2012: 282, description, distribution and biology. Field Guide; Ishikawa 2016: 450, catalogue; Komatsu 2016: 93, description and biology. Field Guide.

#### Materials

- **Type status:**Other material. **Occurrence:** recordedBy: H. Sugahara; individualCount: 1; sex: male; lifeStage: adult; reproductiveCondition: dried specimen; **Taxon:** scientificName: *Pygolampisstriata* Miller, 1940; namePublishedIn: 1940; kingdom: Animalia; phylum: Arthropoda; class: Insecta; order: Hemiptera; family: Reduviidae; genus: Pygolampis; specificEpithet: *striata*; scientificNameAuthorship: Miller, 1940; **Location:** country: Japan; stateProvince: Iwate; municipality: Moriya-shi; locality: Kariyagawa riv.; **Identification:** identifiedBy: Kyosuke Okuda; dateIdentified: 2020; **Event:** samplingProtocol: none specified; eventDate: 21-05-1963; **Record Level:** institutionCode: NIAES; collectionCode: IC; basisOfRecord: PreservedSpecimen
- **Type status:**Other material. **Occurrence:** recordedBy: Y. Uchiyama; individualCount: 1; sex: female; lifeStage: adult; reproductiveCondition: dried specimen; **Taxon:** scientificName: *Pygolampisstriata* Miller, 1940; namePublishedIn: 1940; kingdom: Animalia; phylum: Arthropoda; class: Insecta; order: Hemiptera; family: Reduviidae; genus: Pygolampis; specificEpithet: *striata*; scientificNameAuthorship: Miller, 1940; **Location:** country: Japan; stateProvince: Tochigi; municipality: Noda-chô; locality: Ashikaga-city; **Identification:** identifiedBy: Kyosuke Okuda; dateIdentified: 2020; **Event:** samplingProtocol: none specified; eventDate: 25-09-1981; **Record Level:** institutionCode: NIAES; collectionCode: IC; basisOfRecord: PreservedSpecimen
- **Type status:**Other material. **Occurrence:** recordedBy: M. Fukusawa; individualCount: 1; sex: female; lifeStage: adult; reproductiveCondition: dried specimen; **Taxon:** scientificName: *Pygolampisstriata* Miller, 1940; namePublishedIn: 1940; kingdom: Animalia; phylum: Arthropoda; class: Insecta; order: Hemiptera; family: Reduviidae; genus: Pygolampis; specificEpithet: *striata*; scientificNameAuthorship: Miller, 1940; **Location:** country: Japan; stateProvince: Tokyo; municipality: Nishitama; locality: Fussa; **Identification:** identifiedBy: Kyosuke Okuda; dateIdentified: 2020; **Event:** samplingProtocol: none specified; eventDate: 22-04-1964; **Record Level:** institutionCode: ELTUA; collectionCode: IC; basisOfRecord: PreservedSpecimen
- **Type status:**Other material. **Occurrence:** recordedBy: H. Sotoya; individualCount: 1; sex: female; lifeStage: adult; reproductiveCondition: dried specimen; **Taxon:** scientificName: *Pygolampisstriata* Miller, 1940; namePublishedIn: 1940; kingdom: Animalia; phylum: Arthropoda; class: Insecta; order: Hemiptera; family: Reduviidae; genus: Pygolampis; specificEpithet: *striata*; scientificNameAuthorship: Miller, 1940; **Location:** country: Japan; stateProvince: Tokyo; municipality: Hachiouji-shi; locality: Mt. takao-san; **Identification:** identifiedBy: Kyosuke Okuda; dateIdentified: 2020; **Event:** samplingProtocol: none specified; eventDate: 14-05-1963; **Record Level:** institutionCode: ELTUA; collectionCode: IC; basisOfRecord: PreservedSpecimen
- **Type status:**Other material. **Occurrence:** recordedBy: S. Matsuda; individualCount: 1; sex: female; lifeStage: adult; reproductiveCondition: dried specimen; **Taxon:** scientificName: *Pygolampisstriata* Miller, 1940; namePublishedIn: 1940; kingdom: Animalia; phylum: Arthropoda; class: Insecta; order: Hemiptera; family: Reduviidae; genus: Pygolampis; specificEpithet: *striata*; scientificNameAuthorship: Miller, 1940; **Location:** country: Japan; stateProvince: Tokyo; municipality: Fuchu-shi; **Identification:** identifiedBy: Kyosuke Okuda; dateIdentified: 2020; **Event:** samplingProtocol: none specified; eventDate: 29-08-1946; **Record Level:** institutionCode: NIAES; collectionCode: IC; basisOfRecord: PreservedSpecimen
- **Type status:**Other material. **Occurrence:** recordedBy: Syûji Tachikawa; individualCount: 1; sex: female; lifeStage: adult; reproductiveCondition: dried specimen; **Taxon:** scientificName: *Pygolampisstriata* Miller, 1940; namePublishedIn: 1940; kingdom: Animalia; phylum: Arthropoda; class: Insecta; order: Hemiptera; family: Reduviidae; genus: Pygolampis; specificEpithet: *striata*; scientificNameAuthorship: Miller, 1940; **Location:** country: Japan; stateProvince: Kanagawa; municipality: Atsugi-shi; **Identification:** identifiedBy: Kyosuke Okuda; dateIdentified: 2020; **Event:** samplingProtocol: none specified; eventDate: 29-05-1962; **Record Level:** institutionCode: ELTUA; collectionCode: IC; basisOfRecord: PreservedSpecimen
- **Type status:**Other material. **Occurrence:** recordedBy: Kyosuke Okuda; individualCount: 1; sex: male; lifeStage: adult; reproductiveCondition: dried specimen; **Taxon:** scientificName: *Pygolampisstriata* Miller, 1940; namePublishedIn: 1940; kingdom: Animalia; phylum: Arthropoda; class: Insecta; order: Hemiptera; family: Reduviidae; genus: Pygolampis; specificEpithet: *striata*; scientificNameAuthorship: Miller, 1940; **Location:** country: Japan; stateProvince: Miyazaki; municipality: Mimata-chô, Kitamorokatagun; locality: Miyamura; **Identification:** identifiedBy: Kyosuke Okuda; dateIdentified: 2020; **Event:** samplingProtocol: Light trap; eventDate: 20-05-2013; **Record Level:** institutionCode: PCKO; collectionCode: IC; basisOfRecord: PreservedSpecimen
- **Type status:**Other material. **Occurrence:** recordedBy: Kenji Hidaka; individualCount: 1; sex: female; lifeStage: adult; reproductiveCondition: dried specimen; **Taxon:** scientificName: *Pygolampisstriata* Miller, 1940; namePublishedIn: 1940; kingdom: Animalia; phylum: Arthropoda; class: Insecta; order: Hemiptera; family: Reduviidae; genus: Pygolampis; specificEpithet: *striata*; scientificNameAuthorship: Miller, 194; **Location:** country: Japan; stateProvince: Miyazaki; municipality: Aya-chô; locality: Kitamata; verbatimLocality: Odoubashi; **Identification:** identifiedBy: Kyosuke Okuda; dateIdentified: 2020; **Event:** samplingProtocol: Light trap; eventDate: 27-05-2013; **Record Level:** institutionCode: ELTUA; collectionCode: IC; basisOfRecord: PreservedSpecimen
- **Type status:**Other material. **Occurrence:** recordedBy: Keiichi Takahashi; individualCount: 1; sex: male; lifeStage: adult; reproductiveCondition: dried specimen; **Taxon:** scientificName: *Pygolampisstriata* Miller, 1940; namePublishedIn: 1940; kingdom: Animalia; phylum: Arthropoda; class: Insecta; order: Hemiptera; family: Reduviidae; genus: Pygolampis; specificEpithet: *striata*; scientificNameAuthorship: Miller, 1940; **Location:** islandGroup: Ryukyus; island: Amami-ôshima Is; country: Japan; stateProvince: Kagoshima; municipality: Amami-shi; locality: Sumiyô-son; **Identification:** identifiedBy: Kyosuke Okuda; dateIdentified: 2020; **Event:** samplingProtocol: Light-trap; eventDate: 21-05-2004; **Record Level:** institutionCode: ELTUA; collectionCode: IC; basisOfRecord: PreservedSpecimen
- **Type status:**Other material. **Occurrence:** recordedBy: Akihiro Yoshikawa; individualCount: 1; sex: female; lifeStage: adult; reproductiveCondition: dried specimen; **Taxon:** scientificName: *Pygolampisstriata* Miller, 1940; namePublishedIn: 1940; kingdom: Animalia; phylum: Arthropoda; class: Insecta; order: Hemiptera; family: Reduviidae; genus: Pygolampis; specificEpithet: *striata*; scientificNameAuthorship: Miller, 1940; **Location:** islandGroup: Ryukyus; island: Amami-ôshima Is; country: Japan; stateProvince: Kagoshima; municipality: Uken-son; locality: Uken; decimalLatitude: 28.3115; decimalLongitude: 129.257; geodeticDatum: WGS84; **Identification:** identifiedBy: Kyosuke Okuda; dateIdentified: 2020; **Event:** samplingProtocol: none specified; eventDate: 02-10-2018; **Record Level:** institutionCode: ELTUA; collectionCode: IC; basisOfRecord: PreservedSpecimen
- **Type status:**Other material. **Occurrence:** recordedBy: Kyosuke Okuda; individualCount: 1; sex: male; lifeStage: adult; reproductiveCondition: dried specimen; **Taxon:** scientificName: *Pygolampisstriata* Miller, 1940; namePublishedIn: 1940; kingdom: Animalia; phylum: Arthropoda; class: Insecta; order: Hemiptera; family: Reduviidae; genus: Pygolampis; specificEpithet: *striata*; scientificNameAuthorship: Miller, 1940; **Location:** islandGroup: Ryukyus; island: Amami-ôshima Is; country: Japan; stateProvince: Kagoshima; municipality: Uken-son; locality: Uken; decimalLatitude: 28.18; decimalLongitude: 129.152; geodeticDatum: WGS84; **Identification:** identifiedBy: Kyosuke Okuda; dateIdentified: 2020; **Event:** samplingProtocol: none specified; eventDate: 09-10-2019; **Record Level:** institutionCode: PCKO; collectionCode: IC; basisOfRecord: PreservedSpecimen
- **Type status:**Other material. **Occurrence:** recordedBy: Kyosuke Okuda; individualCount: 1; sex: male; lifeStage: adult; reproductiveCondition: dried specimen; **Taxon:** scientificName: *Pygolampisstriata* Miller, 1940; namePublishedIn: 1940; kingdom: Animalia; phylum: Arthropoda; class: Insecta; order: Hemiptera; family: Reduviidae; genus: Pygolampis; specificEpithet: *striata*; scientificNameAuthorship: Miller, 1940; **Location:** islandGroup: Ryukyus; island: Amami-ôshima Is; country: Japan; stateProvince: Kagoshima; municipality: Uken-son; locality: Uken; decimalLatitude: 28.18; decimalLongitude: 129.152; geodeticDatum: WGS84; **Identification:** identifiedBy: Kyosuke Okuda; dateIdentified: 2020; **Event:** samplingProtocol: none specified; eventDate: 09-10-2019; **Record Level:** institutionCode: PCKO; collectionCode: IC; basisOfRecord: PreservedSpecimen
- **Type status:**Other material. **Occurrence:** recordedBy: Kyosuke Okuda; individualCount: 1; sex: female; lifeStage: adult; reproductiveCondition: dried specimen; **Taxon:** scientificName: *Pygolampisstriata* Miller, 1940; namePublishedIn: 1940; kingdom: Animalia; phylum: Arthropoda; class: Insecta; order: Hemiptera; family: Reduviidae; genus: Pygolampis; specificEpithet: *striata*; scientificNameAuthorship: Miller, 1940; **Location:** islandGroup: Ryukyus; island: Amami-ôshima Is; country: Japan; stateProvince: Kagoshima; municipality: Uken-son; locality: Uken; decimalLatitude: 28.18; decimalLongitude: 129.152; geodeticDatum: WGS84; **Identification:** identifiedBy: Kyosuke Okuda; dateIdentified: 2020; **Event:** samplingProtocol: none specified; eventDate: 09-10-2019; **Record Level:** institutionCode: PCKO; collectionCode: IC; basisOfRecord: PreservedSpecimen
- **Type status:**Other material. **Occurrence:** recordedBy: Kyosuke Okuda; individualCount: 1; sex: female; lifeStage: adult; reproductiveCondition: dried specimen; **Taxon:** scientificName: *Pygolampisstriata* Miller, 1940; namePublishedIn: 1940; kingdom: Animalia; phylum: Arthropoda; class: Insecta; order: Hemiptera; family: Reduviidae; genus: Pygolampis; specificEpithet: *striata*; scientificNameAuthorship: Miller, 1940; **Location:** islandGroup: Ryukyus; island: Amami-ôshima Is; country: Japan; stateProvince: Kagoshima; municipality: Uken-son; locality: Uken; decimalLatitude: 28.18; decimalLongitude: 129.152; geodeticDatum: WGS84; **Identification:** identifiedBy: Kyosuke Okuda; dateIdentified: 2020; **Event:** samplingProtocol: none specified; eventDate: 09-10-2019; **Record Level:** institutionCode: PCKO; collectionCode: IC; basisOfRecord: PreservedSpecimen
- **Type status:**Other material. **Occurrence:** recordedBy: Yoshikawa Akihiro; individualCount: 1; sex: male; lifeStage: adult; reproductiveCondition: dried specimen; **Taxon:** scientificName: *Pygolampisstriata* Miller, 1940; namePublishedIn: 1940; kingdom: Animalia; phylum: Arthropoda; class: Insecta; order: Hemiptera; family: Reduviidae; genus: Pygolampis; specificEpithet: *striata*; scientificNameAuthorship: Miller, 1940; **Location:** islandGroup: Ryukyus; island: Amami-ôshima Is; country: Japan; stateProvince: Kagoshima; municipality: Uken-son; locality: Uken; decimalLatitude: 28.18; decimalLongitude: 129.152; geodeticDatum: WGS84; **Identification:** identifiedBy: Kyosuke Okuda; dateIdentified: 2020; **Event:** samplingProtocol: none specified; eventDate: 12-09-2020; **Record Level:** institutionCode: PCKO; collectionCode: IC; basisOfRecord: PreservedSpecimen
- **Type status:**Other material. **Occurrence:** individualCount: 1; sex: male; lifeStage: adult; reproductiveCondition: dried specimen; **Taxon:** scientificName: *Pygolampisstriata* Miller, 1940; namePublishedIn: 1940; kingdom: Animalia; phylum: Arthropoda; class: Insecta; order: Hemiptera; family: Reduviidae; genus: Pygolampis; specificEpithet: *striata*; scientificNameAuthorship: Miller, 1940; **Location:** islandGroup: Malay Archipelago; island: Kalimantan Is.; country: Indonesia; stateProvince: East Kalimantan; municipality: Kurao, Grogot; locality: Sungai Nangka; verbatimElevation: 255 m; **Identification:** identifiedBy: Kyosuke Okuda; dateIdentified: 2020; **Event:** samplingProtocol: none specified; eventDate: 30-12-2000; **Record Level:** institutionCode: CMB; collectionCode: IC; basisOfRecord: PreservedSpecimen

#### Description

**Figure 1.**
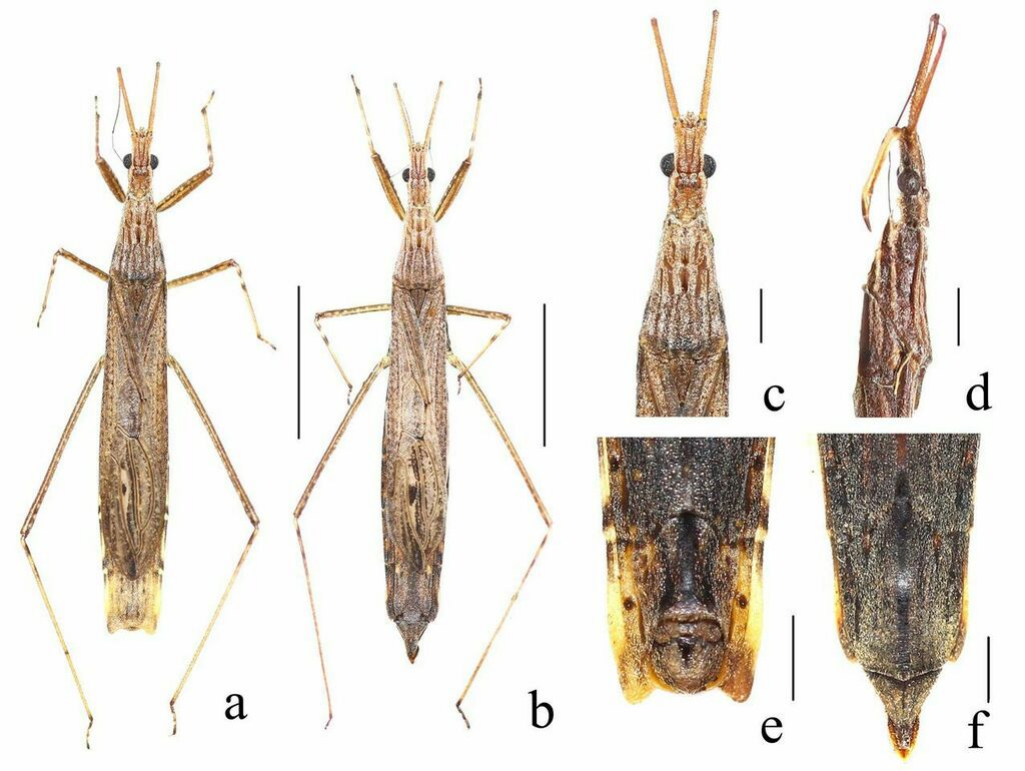
*Pygolampisstriata* Miller, 1940. a–b: dorsal view, a. male, b. female, c–d: male head and pronotum, c. dorsal view, d. lateral view, e–f: apex of abdomen, e. male, f. female. Scale bar: a, b 5.0 mm; c–f 1.0 mm.

**Male. Colouration.** Body generally whitish-brown to pale brown on dorsal side (Fig. 1a). Head pale brown on dorsal side and ventral side (Fig. 1c); head dark brown on lateral side, with longitudinal blackish lines (Fig. 1d). Compound eyes black; ocelli reddish-brown. Antennal segment I pale brown, II-IV dark brown. Visible labial segments I–II pale brown, III dark brown. Thorax dark brown with longitudinal whitish-brown lines on lateral side and dark brown on ventral side. Hemelytra generally whitish-brown to pale brown, with blackish spot in vein of cubitus. Abdomen (Fig. 1e) pale brown on dorsal side with sparse brownish spots, dark brown on ventral side with sparse yellow spots; connexiva yellow with brownish spots on segments II-VI. Legs whitish-brown to pale brown; profemur with irregular markings on outer surface; pro- and mesotibia with dark brown annulations on basal third and apex; tarsus dark brown.

**Vestiture.** Head densely covered with whitish decumbent pubescence, interantennal tubercle with setae at apex. Eye glabrous. Antennal segment I with decumbent pubescence; segments II–IV with whitish sub-erect setae, as long as the diameter of each segment. Labium with sparse short blackish sub-erect setae, as long as the diameter of half of each segment. Pronotum with densely covered whitish pubescence, three longitudinal rows of whitish pubescence rows on lateral side. Scutellum without pubescence. Hemelytra with whitish pubescence on the corium and veins. Abdomen with fine pubescence. Hind leg tibia with setae subapically; mid- and hind leg tibia with dense setae uniformly.

**Figure 2.**
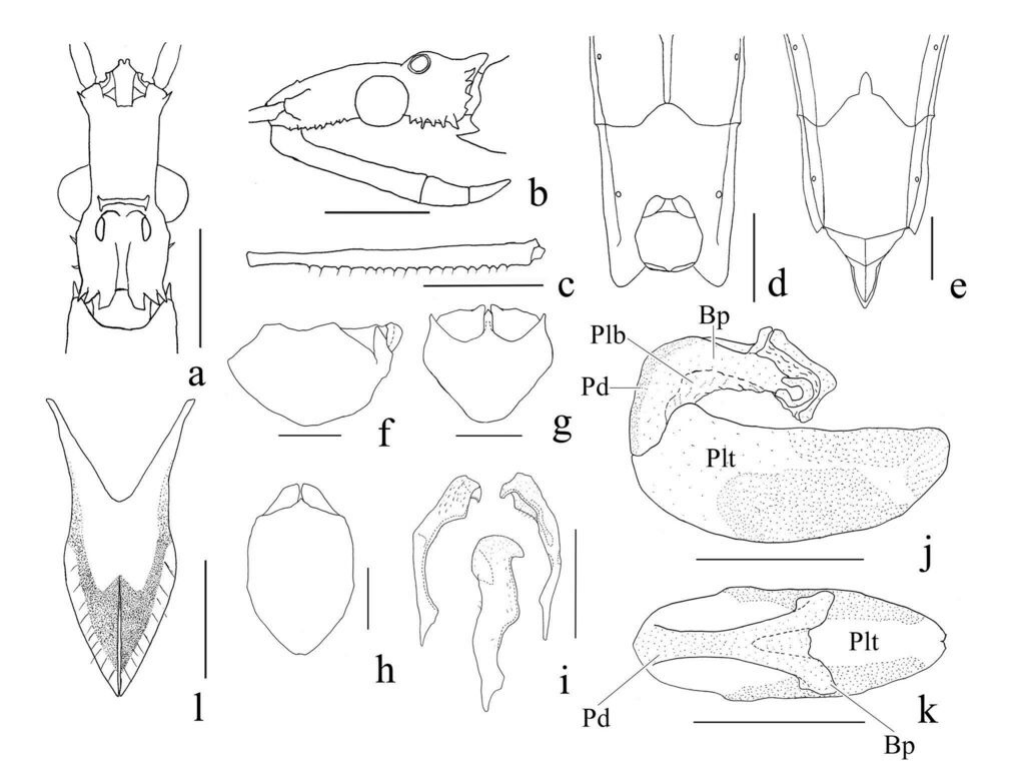
*Pygolampisstriata* Miller, 1940, a–b: head, a. dorsal view, b. lateral view, c. antennal segment I, d–e: apex of abdomen ventral view, d. male, e. female, f–h: pygophore, f. lateral view omitted on left paramere, g. caudal view, h. dorsal view, i. parameres, j–k: phallus, j. lateral view, k. dorsal view (Lettering: Bp = Basal plate; Pd = pedicel; Plb = phallobase; Plt = phallotheca), l styloides. Setae omitted in a, b, d–k. Scale bar: a–e, 1.0 mm, f–l, 0.5 mm.

**Structure.** Body elongate. Head (Fig. 2a, b) oblong, approximately 1.7 times long as eye width, approximately 0.8 times long as pronotum; ante-ocular portion approximately 1.25 times long as postocular portion, with glabrous lines in the basal half, diverging apically; ante-ocular part with four glabrous lines on dorsum. Compound eye protuding laterally, hemispherical; ocelli upwardly prominent. Interantennal tubercle diverging apically, upwardly prominent. Postocular tubercule and lateroventral tubercle short, irregular. Antenna (Fig. 2c) cylindrical; scape (antennal segment I) approximately 1.0–1.1 times as long as head length; approximate proportion of segments I to IV 9: 10: 1.9: 5. Labium approximate proportion of visible segments I to III 1: 0.3: 0.2; visible segment I straight, segments II and III curved. Pronotum long, trapezoidal on longitudinal sulcate in middle, widened posteriad, approximately 1.6 times as long as its maximum width; postocular lobe elevated, with six longitudinal carina dorsally; posterolateral angle elevated, obtuse; ante-ocular propleural spines short, robust, approximately 0.5 times as long as eye width. Scutellum longer than wide, with medial oval depression. Hemelytra macropterous, extending beyond base of the abdominal segment VII. Abdomen (Fig. 2d), segment V widest, posterior angle projecting backwards, weakly concave at middle. Femur longer than tibia, hind leg femur extending to level of segment VII.

**Male genitalia.** Pygophore (Fig. 2f–h) elongate, rounded dorsal view; dorsally membranous, anteroventral surface granulated; median process short, robust, lateral side depressed; parameres (Fig. 2i) symmetrical, apical portions relatively broad, weakly curved, twisting; phallus shown as Fig. 2j–k; phallobase membranous, boundary with pedicel unclear; phallotheca oval when viewed from above in dorsal view, approximately 1.8 times as long as pedicel, struts invisible from outside.

**Female.** General aspect as in male (Fig. 1b), but different as follows: ventral posterior margin of abdominal segment VI with strongly incised middle, abdominal apical segment strongly produced (Fig. 1f, Fig. 2e); styloides (Fig. 2l) incised in half of the apical part, with setae on margin; posterior femur extends beyond abdominal segment VI, apex of posterior femora not reaching abdominal segment VII.

**Measurements**: [in mm, male (n = 7)/female (n = 9)]. Body length 15.80–16.50/17.50–19.50; head length 2.05–2.25/2.00–2.35; length of anteocular part 0.85–0.90/0.85–1.00; length of postocular part 0.65–0.75/0.65–0.75; lengths of antennal segments I 2.25–2.35/2.40–2.95, II 2.25–2.60/2.30–2.80, III 0.40–0.50/0.40–0.60, IV 0.80–1.30/0.90–1.25; lengths of visible labial segments I 1.35–1.60/1.50–1.75, II 0.45–0.55/0.40–0.50, III 0.35–0.40/0.30–0.40; length of pronotum (including propleural spines) 3.00–3.30/3.10–3.75; maximum width of thorax 1.75–1.95/1.75–2.10; length of scutellum 0.75–1.00/0.80–1.05; maximum width of scutellum 0.50–0.60/0.50–0.75; length of the hemelytron 9.1–10.0/10.0–11.5; length of fore leg femur 3.00–3.20/3.00–3.20, tibia 2.70/2.70, tarsus 0.65–0.70/0.70; of mid-leg femur 3.40–3.50/3.20–4.00, tibia 2.75/2.75–3.20, tarsus 0.75–0.80/0.65–0.80; of hind leg femur 7.50–8.00/7.50–8.00, tibia 7.50–8.00/7.50–8.00, tarsus 0.90–1.00/0.90–1.00.

#### Diagnosis

This species can be distinglished from other species of *Pygolampis* by the following set of characters: body length 15.80-16.50 mm in the male, 17.50-19.50 mm in female, generally whitish-brown to pale brown, with densely covered whitish decumbent pubescence on dorsal side; pronotum with three longitudinal rows of whitish pubescence on lateral side; abdomen dark brown on ventral side with sparse yellow spots; abdominal segment VII posterior angle shown as Fig. 1e.

In general appearance, *Pygolampisstriata* Miller, 1940 is very similar to *P.bidentata* (Gozze, 1977), but can be distinguished from the latter by a combination of the following characters: generally whitish-brown to pale brown (in *P.bidentata*, generally dark brown); thorax with three longitudinal rows of whitish pubescence on lateral side (in *P.bidentata*, thorax without three longitudinal rows); abdomen dark brown on ventral side with sparse yellow spots (in *P.bidentata*, abdomen without sparse yellow spots ventrally). Additionally, similar distribution to *P.foeda* Stål, 1859 in Japan, but can be distinguished from the latter by a combination of the following characteristics: pale brown body and thorax with three longitudinal rows of whitish pubescence on lateral side. (in *P.foeda*, generally reddish-brown-dark brown, thorax without three longitudinal rows of whitish pubescence on lateral side); scape approximately 1.0–1.1 times as long as head length (in *P.foeda*, scape approximately 1.2–1.4 times as long as head length); posterior angle projecting backwards, weakly concave at middle. (in *P.foeda*, posterior angle projecting backwards, slightly deep concave at middle).

#### Distribution

Japan: Honshu, Kyusyu, Ryukyus [Amami-ôshima Is.], Indonesia [East Kalimantan], Malaysia [Sandakan].

#### Conservation

In Honshu, Japan, there is no specimen of the collection since 1981, indicating the likelihood that the species might be extinct in the region.

## Discussion

Some studies have evaluated heteropteran fauna in Amami-ôshima (Okuda et al. 2020, Souma 2018, Tomokuni 1989); however, *P.striata* was not found until recently. This may be because Stenopodainae is found mainly in grasslands and on the surface of herbaceous vegetation; moreover, it is difficult to collect the specimens using the sweeping or beating method. Most Stenopodainae members have been collected at night, with almost no record of direct observations of their behaviour during the day (Schuh and Weirauch 2020).

Therefore, the collection of specimens of this species using methods other than light traps is difficult. Even with light traps, it is difficult to target and collect certain species; *P.striata* and many Stenopodainae members require a special collection method. Ishikawa and Miyamoto (2012) proposed the "Gasa-Gasa collecting method" for the collection of these ground-dwelling Reduviidae members. This method involves searching the ground surface at the base of gramineous plants (“Gasa-Gasa" is an onomatopoeic sound produced while searching for the species at the base of gramineous plants). I successfully collected *P.striata* from the surface of dried Poaceae grasslands in Amami-ôshima using this method (Fig. 3). This method has also been successfully employed for the collection of other Stenopodainae species (I also collected *Oncocephalusgermari* Reuter, 1882; *O.heissi* Ishikawa, Cai et Tomokuni, 2006; and *Sastrapadaoxyptera* Bergroth, 1922 in Amami-ôshima). Hence, for the aforementioned reasons, a survey using the "Gasa-Gasa collecting method" might reveal the current distribution of Stenopodainae members, including *P.striata* in Asia.

## Supplementary Material

XML Treatment for Pygolampis striata

## Figures and Tables

**Figure 3a.**
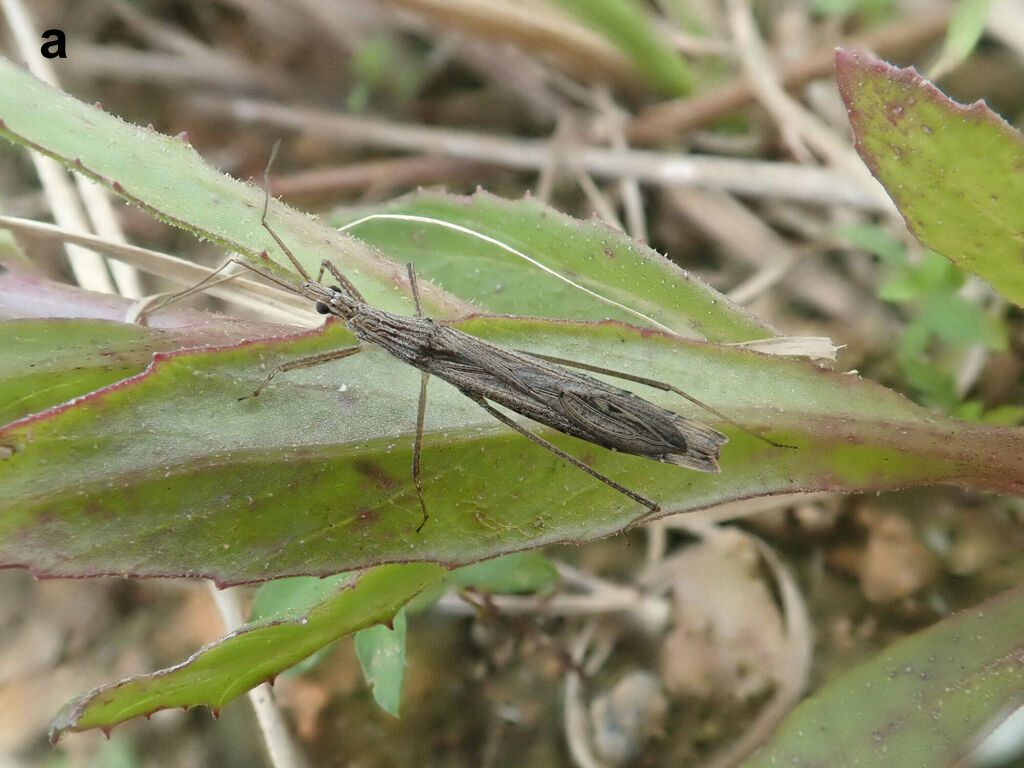
Living male

**Figure 3b.**
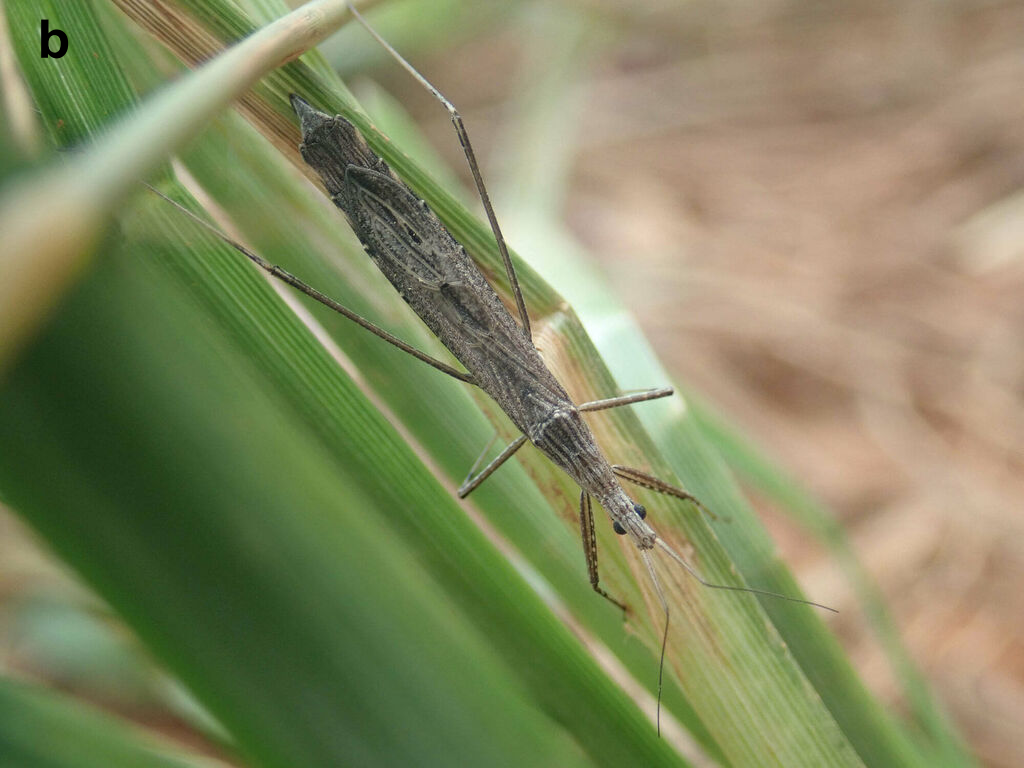
Living female

**Figure 3c.**
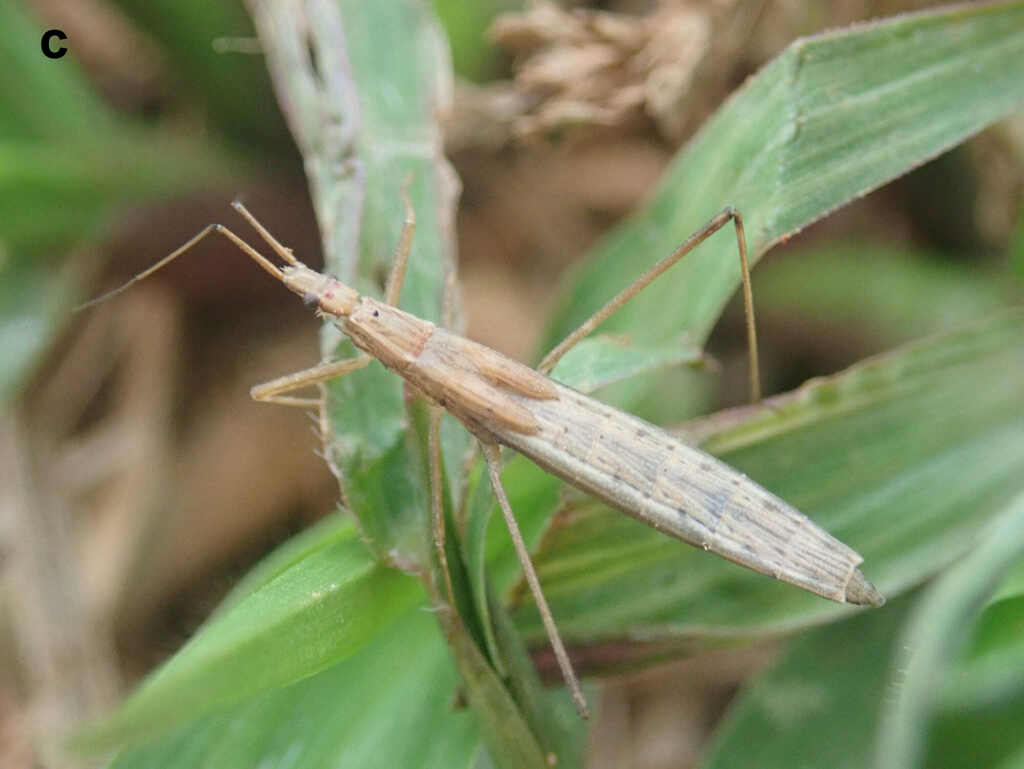
5th instar nymph

**Figure 3d.**
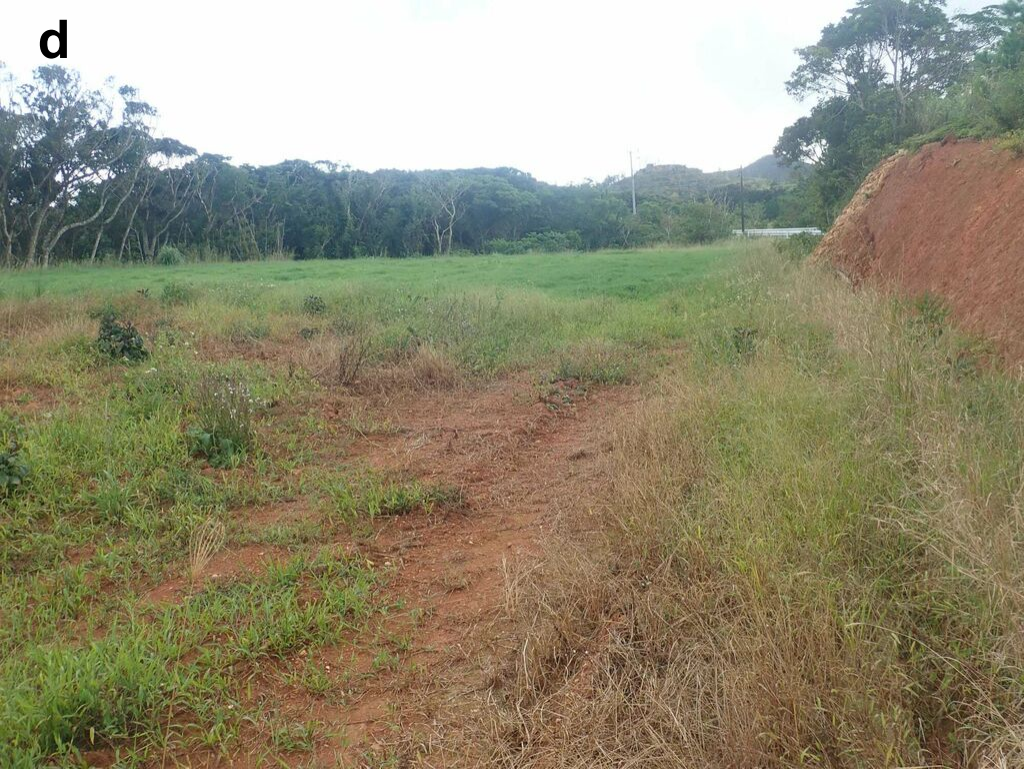
Habitat
